# Brain Tissue Oxygenation, Cardiac Complications, and Haemodynamic Interactions After Aneurysmal Subarachnoid Haemorrhage: A Systematic Review

**DOI:** 10.7759/cureus.117995

**Published:** 2026-10-15

**Authors:** Georgia Chatzopoulou, Christos Voucharas, Polychronis Antonitsis, Thomas Gkosios, Ioannis Dravopoulos, Nikolaos Georgakoulias

**Affiliations:** 1 School of Medicine, Aristotle University of Thessaloniki, Thessaloniki, GRC; 2 Cardiothoracic Surgery Department, Aristotle University of Thessaloniki, Thessaloniki, GRC; 3 Cardiothoracic Department, AHEPA University Hospital, Aristotle University of Thessaloniki School of Medicine, Thessaloniki, GRC; 4 Cardiology Department, AHEPA University Hospital, Aristotle University of Thessaloniki School of Medicine, Thessaloniki, GRC; 5 Neurosurgery Department, Athens State Hospital "Georgios Gennimatas", Athens, GRC

**Keywords:** aneurysmal subarachnoid haemorrhage, brain tissue oxygen tension, cardiac complications, cardiocerebral interaction, cerebral perfusion pressure, haemodynamic monitoring, multimodal neuromonitoring, pbto₂

## Abstract

Brain tissue oxygen tension (PbtO₂) monitoring is increasingly used as a component of multimodal neuromonitoring in patients with poor-grade aneurysmal subarachnoid haemorrhage (aSAH). However, it remains uncertain whether PbtO₂ abnormalities predict early cardiac complications and how systemic haemodynamic and cardiorespiratory factors influence regional cerebral oxygenation. This systematic review aimed to determine whether invasively measured PbtO₂ is associated with subsequent early cardiac complications or haemodynamic instability of cardiovascular origin after aSAH and to synthesise evidence on the effects of systemic haemodynamic and selected cardiorespiratory variables and interventions on PbtO₂. PubMed/MEDLINE, Web of Science, Scopus, CENTRAL, Europe PMC, and ClinicalTrials.gov were searched, together with backward citation searching, to identify studies of adults with aSAH who underwent invasive PbtO₂ monitoring. No restrictions on publication date or language were applied. The review was conducted and reported in accordance with the Preferred Reporting Items for Systematic Reviews and Meta-Analyses (PRISMA) 2020, and methodological quality was appraised using design-specific Joanna Briggs Institute critical appraisal tools. Owing to substantial clinical and methodological heterogeneity, findings were synthesised narratively.

The searches identified 961 records, of which 21 publication reports published between 2005 and 2026 met the eligibility criteria. None directly evaluated whether antecedent PbtO₂ abnormalities or cumulative hypoxic burden predicted subsequent early cardiac complications. The available evidence predominantly addressed the reverse relationship, examining how systemic physiological variables and therapeutic interventions affected regional brain tissue oxygenation. Lower mean arterial pressure or cerebral perfusion pressure was frequently associated with lower PbtO₂, whereas controlled arterial pressure augmentation often improved oxygenation. Responses were heterogeneous and appeared to depend on baseline oxygenation, cerebrovascular autoregulatory status, probe location, and local cerebral pathology. Fluid administration produced larger increases in PbtO₂ when accompanied by an increase in cardiac index. Respiratory, pharmacological, and osmotic interventions had variable effects, reflecting their combined systemic, cerebral, and intracranial consequences. Lower PbtO₂ or impaired oxygen reactivity was associated in some reports with metabolic crisis, delayed cerebral infarction, spreading depolarisation, mortality, or unfavourable neurological outcome. However, independent prognostic value was not consistently demonstrated. Meta-analysis was not appropriate because of heterogeneity in study designs, interventions, physiological thresholds, analytical methods, repeated observations, and potentially overlapping cohorts.

Current evidence does not establish PbtO₂ as a predictor of early cardiac complications after aSAH. PbtO₂ is a regional and context-dependent physiological measure influenced by interacting systemic, cerebral, respiratory, and intracranial factors. It should therefore be interpreted as part of multimodal neuromonitoring rather than according to a universal haemodynamic or oxygen threshold. Prospective multicentre studies incorporating synchronised PbtO₂ and cardiac monitoring, standardised hypoxic-burden metrics, and temporally adjudicated cardiac outcomes are required.

## Introduction and background

Aneurysmal subarachnoid haemorrhage (aSAH) is a severe haemorrhagic stroke associated with substantial mortality, long-term neurological disability, and intensive care use [1-3]. Early brain injury and secondary cerebral insults may coexist with cardiac and systemic haemodynamic complications caused partly by sympathetic activation and catecholamine excess. These complications include myocardial injury, QTc prolongation, arrhythmias, ventricular dysfunction, neurogenic stress cardiomyopathy, pulmonary oedema, and haemodynamic instability [4-6]. By reducing cardiac output or systemic oxygen delivery, they may further compromise cerebral perfusion, particularly when cerebrovascular autoregulation is impaired.

Invasive brain tissue oxygen tension (PbtO₂) monitoring is used within multimodal neuromonitoring in selected patients with poor-grade aSAH [1,7-10]. It reflects the local balance between cerebral oxygen delivery and consumption in the tissue surrounding the probe. Although values below 20 mmHg are commonly used to define brain tissue hypoxia, PbtO₂ is regional and context-dependent. Its interpretation is influenced by probe location, local pathology, cerebral blood flow, intracranial pressure, cerebral perfusion pressure, autoregulation, haemoglobin concentration, arterial oxygenation, carbon dioxide tension, mean arterial pressure, and cardiac output [7-11].

This physiological interdependence creates two related but distinct questions. First, it remains uncertain whether antecedent PbtO₂ abnormalities predict subsequent early cardiac complications or haemodynamic instability of cardiovascular origin. Second, systemic haemodynamic and cardiorespiratory factors may alter PbtO₂, but the relevant evidence is dispersed across heterogeneous observational and short-term interventional studies. Previous reviews have focused mainly on cerebral hypoxia, delayed cerebral ischaemia, neurological outcome, or systemic haemodynamic monitoring rather than temporally ordered prediction of cardiac events [10,11].

The primary objective of this systematic review was to determine whether invasively measured PbtO₂ is associated with the subsequent development of early cardiac complications or haemodynamic instability of cardiovascular origin in adults with aSAH during the acute neurocritical care period. The secondary objective was to synthesise evidence on the effects of cardiac performance, systemic haemodynamic factors, and selected cardiorespiratory variables and interventions on PbtO₂.

## Review

Methods

Reporting Standard and Protocol

This systematic review was conducted and reported in accordance with the Preferred Reporting Items for Systematic Reviews and Meta-Analyses (PRISMA) 2020 statement [12]. The eligibility criteria, outcomes, operational definitions, and analytical framework were finalised before the updated literature searches and the rescreening of all retrieved records. No separate review protocol was prepared, and the review was not prospectively registered.

Eligibility Criteria

Reports were eligible if they included adults aged 18 years or older with confirmed aSAH. Reports involving mixed subarachnoid haemorrhage populations or broader acute brain injury cohorts were eligible only when data for participants with aSAH were reported separately and could be directly extracted. Studies limited to traumatic, non-aneurysmal, perimesencephalic, or unspecified subarachnoid haemorrhage were excluded, as were reports in which the aSAH subgroup could not be separated from other diagnostic populations.

Eligible reports were required to use invasive intraparenchymal monitoring of brain tissue oxygen tension and to report PbtO₂, PtiO₂, PBrO₂, or an equivalent invasively measured parameter. Reports assessing cerebral oxygenation exclusively using non-invasive methods, including near-infrared spectroscopy or regional cerebral oxygen saturation, were excluded. Reports limited to jugular venous oxygen saturation, cerebral perfusion imaging, cerebral microdialysis, or other surrogate measures without extractable invasive brain tissue oxygen data were also excluded.

For the primary review question, eligible reports had to evaluate the relationship between invasively measured brain tissue oxygen tension and at least one early cardiac complication or episode of haemodynamic instability attributable to cardiovascular dysfunction. Outcomes of interest included clinically significant arrhythmias, QTc prolongation, acute myocardial injury, new-onset left ventricular systolic dysfunction, regional wall-motion abnormalities, neurogenic stress cardiomyopathy, cardiogenic pulmonary oedema, and haemodynamic instability of cardiovascular origin.

For the secondary review question, eligible exposures or interventions included cardiac output or cardiac index, intravascular volume status, fluid responsiveness, fluid administration, mean arterial pressure, cerebral perfusion pressure, arterial pressure manipulation, vasopressor or inotropic therapy, induced hypertension, and pharmacological or osmotic interventions with measurable systemic, cerebral, or intracranial haemodynamic effects. Selected cardiorespiratory variables and interventions were also eligible when they could plausibly modify brain tissue oxygenation. These included positive end-expiratory pressure, prone positioning, inhaled pulmonary vasodilator therapy, arterial oxygenation, and controlled manipulation of arterial carbon dioxide tension. Studies in which a sedative or anaesthetic agent constituted the principal intervention were excluded, although sedation was retained as a potential confounder or co-intervention within broader multimodal treatment protocols.

Eligible designs included randomised and non-randomised interventional studies, prospective and retrospective cohort studies, controlled and uncontrolled before-after physiological studies, repeated-intervention studies, and case series reporting extractable group-level clinical or physiological data. Only full-text reports of human studies presenting original findings were included. Systematic reviews, scoping reviews, meta-analyses, guidelines, narrative reviews, editorials, commentaries, animal or laboratory studies, individual case reports, study protocols, trial registry entries without published results, and conference abstracts without an accompanying full report were excluded. No restrictions on publication date or language were applied.

Outcomes and Operational Definitions

The primary outcome was the reported association between invasively measured brain tissue oxygen tension and the subsequent occurrence of an early cardiac complication or an episode of haemodynamic instability of cardiovascular origin. Early events were operationally defined as those occurring within seven days of admission to the intensive care unit. When a report used a different temporal reference, such as the number of days after haemorrhage onset or the entire intensive care unit stay, the original time frame was retained and explicitly reported.

A report was considered to provide prognostic evidence only when the PbtO₂ measurement or exposure period clearly preceded the cardiac outcome. Contemporaneous or cross-sectional associations and immediate intervention-related responses were retained as mechanistic evidence but were not interpreted as demonstrating temporal prediction. Vasoactive treatment administered solely for protocolised cerebral perfusion pressure augmentation or the treatment of delayed cerebral ischaemia was not classified as evidence of cardiovascular dysfunction unless systemic circulatory failure, myocardial dysfunction, clinically significant arrhythmia, or another cardiovascular indication was documented. Individual cardiac outcomes were considered separately, and no de novo composite cardiac outcome was derived from published aggregate data.

Secondary physiological outcomes comprised the reported direction and magnitude of changes in brain tissue oxygen tension associated with eligible systemic haemodynamic or cardiorespiratory variables and interventions. For interventional, repeated-intervention, and before-after studies, extracted outcomes included baseline and post-intervention PbtO₂ values, absolute or relative changes, measurement intervals, duration of response, and corresponding systemic, cerebral, respiratory, or intracranial physiological changes.

Secondary clinical outcomes included delayed cerebral ischaemia, vasospasm, cerebral infarction, mortality, neurological or functional outcome, duration of mechanical ventilation, and intensive care unit length of stay. Delayed cerebral ischaemia was classified according to the consensus definition proposed by Vergouwen et al. when this definition was used by the original investigators. Otherwise, the original study definition was retained [13]. Brain tissue oxygen measures were extracted using the terminology, thresholds, and definitions reported by the original investigators. These included absolute or serial values, intervention-related changes, study-defined brain tissue hypoxia, cumulative or time-weighted measures of hypoxic burden, and PbtO₂-derived indices of oxygen reactivity or autoregulation. A PbtO₂ threshold below 20 mmHg was used as the principal reference for organising the narrative synthesis because it was the most commonly applied definition of brain tissue hypoxia in the included literature and in the broader neurocritical care context [9,10]. Studies using alternative thresholds or analysing PbtO₂ as a continuous variable were retained without retrospective conversion or harmonisation. No de novo measure of hypoxic burden was calculated. Study-specific definitions, thresholds, analytical units, exposure periods, and follow-up time points were retained as reported.

Information Sources and Search Strategy

MEDLINE via PubMed, Web of Science, Scopus, the Cochrane Central Register of Controlled Trials (CENTRAL), Europe PMC, and ClinicalTrials.gov were searched without restrictions on publication date or language. The searches were initially completed in July 2026 and were updated on 10 October 2026. The updated searches identified no additional records. The search combined two principal concept groups: terms for subarachnoid haemorrhage and terms for invasive brain tissue oxygen monitoring. Relevant British and American spelling variants, abbreviations, and alternative terminology for PbtO₂ were included. Cardiac, haemodynamic, and cardiorespiratory terms were not used as a mandatory third concept block. The corresponding eligibility criteria were applied during screening to avoid excluding physiological studies in which eligible exposures were not stated explicitly in the title or abstract. Search syntax and field restrictions were adapted to each information source. Complete search strategies, search dates, and raw record counts are provided in Appendix A.

The reference lists of potentially eligible reports and relevant evidence syntheses were examined by backward citation searching on 24 July 2026. Forward citation searching was conducted using the OpenAlex scholarly bibliographic database and citation index on 10 October 2026 using all 21 included publication reports as seed reports. This search identified 808 citing records. After deduplication against the existing review dataset, 715 additional records underwent title and abstract screening. No additional eligible report was identified.

Record Management and Study Selection

Retrieved records were imported into Zotero (Corporation for Digital Scholarship, Vienna, Virginia, United States) for reference management and initial duplicate identification. Screening decisions, reasons for exclusion, data extraction, critical appraisal, and the review audit trail were documented in structured Microsoft Excel workbooks (Microsoft Corporation, Redmond, Washington, United States). Records were deduplicated hierarchically using normalised unique identifiers and bibliographic fields, with near-matching titles reviewed manually. Deduplication was performed at the bibliographic-record level. Distinct publication reports were retained even when they potentially described overlapping patient populations.

A single reviewer conducted deduplication, title and abstract screening, full-text eligibility assessment, data extraction, and critical appraisal using predefined criteria. Records identified through the updated searches and forward citation searching were deduplicated against the existing dataset and screened using the same eligibility criteria. Reports were advanced to full-text assessment when they appeared potentially eligible or when the available information was insufficient to support exclusion. Full texts were sought through legally accessible publisher, bibliographic, institutional, and author-sharing sources. Unavailable reports were classified as not retrieved. The 17 reports sought for retrieval but not obtained in full text are presented in Appendix B. Each retrieved report was assessed against all eligibility domains, and one primary reason was recorded for each full-text exclusion. Reports were not excluded on the basis of methodological quality. Reports identified through backward citation searching underwent the same assessment process. Extracted numerical and methodological information was rechecked against the corresponding full-text reports before synthesis.

Data Extraction and Cohort-Overlap Assessment

Data were extracted at the level of the individual publication report using standardised Microsoft Excel forms developed for this review. Extracted items included bibliographic and design characteristics, population characteristics and neurological severity, total sample size and the number of participants contributing PbtO₂ data, monitoring device, probe location, monitoring period, hypoxia threshold, eligible exposures and interventions, physiological and clinical outcomes, statistical methods, effect estimates, confidence intervals, p-values, and major study-specific limitations.

When PbtO₂ monitoring was available only for a subgroup, the PbtO₂-specific analytical denominator was used to interpret oxygen-related findings. Numerical results were retained in the units, thresholds, analytical scales, and time intervals reported by the original investigators. No unpublished values were inferred, no data were imputed, and information that was not reported or could not be extracted separately was recorded accordingly. Adjusted estimates were preferred when available. For studies involving repeated interventions or multiple observations within the same participants, the original unit of analysis was preserved, and the resulting non-independence was considered during interpretation.

Potential overlap between patient populations was assessed by comparing institutions, recruitment periods, investigator groups, clinical populations, neuromonitoring programmes, sample sizes, and study characteristics. Reports were conservatively classified as having a high likelihood of overlap, possible overlap, or no identified overlap. Potentially overlapping reports were retained when they presented distinct exposures, interventions, analyses, or outcomes but were not interpreted as providing independent replication. Because overlap at the individual-patient level could not be determined reliably, participant numbers were not summed across reports.

Critical Appraisal

All included reports underwent design-specific critical appraisal using Joanna Briggs Institute (JBI) instruments. Cohort reports were assessed using the revised JBI tool for cohort studies, non-randomised interventional and before-after physiological reports were assessed using the revised JBI tool for quasi-experimental studies, and case series were assessed using the JBI case-series tool [14-16]. The instrument was selected according to the study design and the analytical component that generated the evidence relevant to this review, rather than solely according to the terminology used by the original investigators.

Judgements were based on the information reported in the full text. Items that were not applicable to a particular design were recorded as such, whereas insufficiently reported information was classified as unclear. To facilitate presentation across study designs, item-level findings were organised descriptively into five overarching domains: participant selection and comparability, identification and measurement of the exposure or intervention, control of confounding and temporal trends, outcome measurement and follow-up, and statistical analysis. These domains were used solely as a reporting framework and do not represent official JBI categories, validated risk-of-bias ratings, or numerical scores. No aggregate score or threshold for exclusion was applied. Appraisal findings were incorporated into the interpretation of individual reports and the overall synthesis. Study-level domain judgments, appraisal decisions, and key methodological limitations are presented below.

Synthesis Methods

The individual publication report was the unit of synthesis. A structured narrative synthesis was undertaken because the included reports differed substantially in study design, population, exposure or intervention, PbtO₂ measure, physiological threshold, measurement interval, analytical unit, and outcome. Evidence was organised according to the two review questions and grouped into five clinically coherent domains: (1) systemic arterial pressure, cerebral perfusion pressure, and autoregulation; (2) cardiac output, fluid responsiveness, and multimodal physiological optimisation; (3) respiratory and gas-exchange interventions; (4) pharmacological and osmotic interventions; and (5) neurological and clinical outcomes.

For the primary review question, reports were examined to determine whether PbtO₂ measurements clearly preceded a subsequent cardiac outcome. Contemporaneous associations and immediate intervention-related responses were treated as mechanistic rather than prognostic evidence. Within each domain, findings were compared according to the direction and magnitude of the association or response, statistical precision, methodological limitations, potential confounding, and possible cohort overlap. Where available, the synthesis reported the magnitude and direction of associations together with effect estimates and 95% confidence intervals, distinguished adjusted from unadjusted analyses, and did not interpret non-significant results as evidence of no association. Adjusted estimates were preferred when available, and missing effect estimates were not reconstructed.

Meta-analysis was not undertaken because sufficiently comparable effect estimates were unavailable. Heterogeneity in interventions, physiological targets, PbtO₂ definitions, analytical time scales, outcome measures, and units of analysis, together with repeated non-independent observations and potential cohort overlap, precluded reliable quantitative pooling. Formal subgroup analyses, meta-regression, and statistical sensitivity analyses were therefore not performed.

Formal statistical assessment of reporting bias was not undertaken because the number of reports within each evidence domain was small and the evidence was highly heterogeneous. Potential selective reporting was considered qualitatively during data extraction, critical appraisal, and synthesis. A formal framework for assessing certainty of evidence was not applied. Confidence in the findings was evaluated narratively according to study design, methodological limitations, sample size, precision, consistency, directness, residual confounding, non-independent observations, and cohort overlap. Findings from small uncontrolled studies, incompletely reported analyses, or potentially overlapping cohorts were interpreted as exploratory.

Results

Study Selection

The searches identified 1,769 records: 938 from bibliographic databases (PubMed/MEDLINE: n=221; Web of Science: n=271; Scopus: n=263; CENTRAL: n=10; Europe PMC: n=173), 22 from ClinicalTrials.gov, one through backward citation searching, and 808 through forward citation searching. After the removal of 645 duplicates, 1,124 records underwent title and abstract screening, of which 1,079 were excluded. Forty-five reports were sought for retrieval. Seventeen could not be obtained through the available legal access routes and were classified as reports not retrieved (Appendix B). The remaining 28 reports were assessed for eligibility. Seven reports were excluded after full-text assessment: three did not examine an eligible relationship between a systemic haemodynamic or selected cardiorespiratory exposure and PbtO₂, including reports limited to local cerebral interventions outside the prespecified scope, and four included mixed or non-separable populations without independently extractable data for adults with aSAH. A total of 21 publication reports met the eligibility criteria and were included in the review (Figure 1).

**Figure 1:**
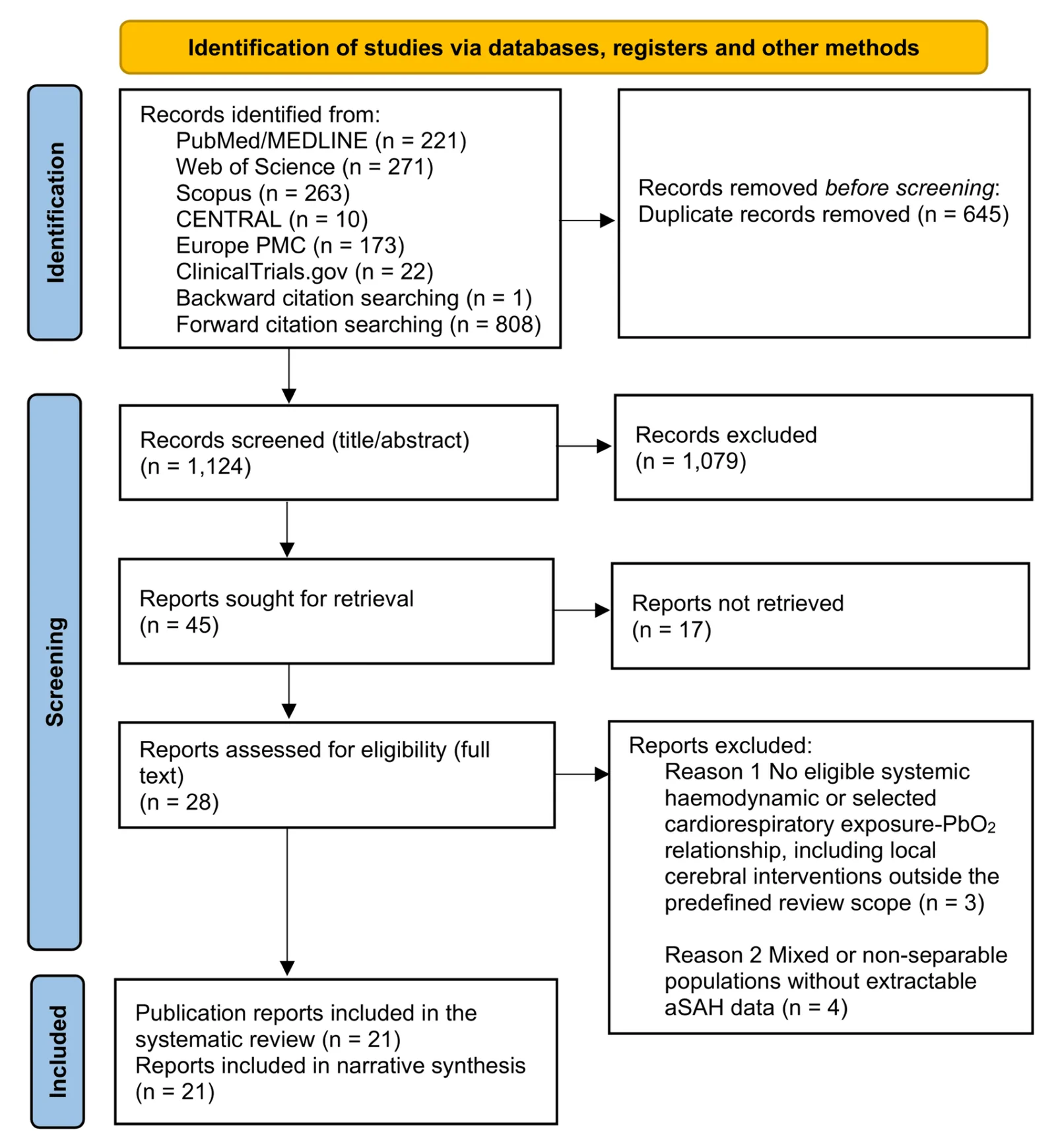
PRISMA 2020 flow diagram of study identification and selection Flow diagram illustrating study identification, screening, full-text eligibility assessment, and inclusion of publication reports in the systematic review. PRISMA: Preferred Reporting Items for Systematic Reviews and Meta-Analyses; CENTRAL: Cochrane Central Register of Controlled Trials; aSAH: aneurysmal subarachnoid haemorrhage

Characteristics of the Included Reports

The 21 included publication reports were published between 2005 and 2026 [17-37]. Eight were appraised as cohort studies, 10 as quasi-experimental studies, and three as case series. No randomised controlled trial met the eligibility criteria. Sample sizes ranged from five to 320 participants. In most reports, the total sample size and the number of participants contributing invasive brain tissue oxygen measurements were identical. In one pooled multimodal monitoring cohort, PbtO₂ data were available only for a subset of participants, and the PbtO₂-specific denominator was not reported separately.

The studies were conducted in Europe and the United States and predominantly enrolled patients with poor-grade aSAH, an impaired neurological examination, delayed cerebral ischaemia, vasospasm, respiratory failure, systemic hypotension, or another indication for invasive multimodal neuromonitoring. The evidence base consisted mainly of observational cohorts and short-term physiological intervention studies rather than comparative effectiveness trials.

The principal exposures and interventions included spontaneous or induced changes in mean arterial pressure and cerebral perfusion pressure, induced hypertension, vasopressor therapy, changes in cardiac index in response to fluid administration, multimodal PbtO₂-directed optimisation, prone positioning, inhaled nitric oxide, oral or intra-arterial nimodipine, intravenous diclofenac, and hypertonic saline. Several reports also examined PbtO₂-derived oxygen-reactivity indices and their associations with regional hypoperfusion, spreading depolarisation, delayed cerebral infarction, and neurological outcome.

Licox (Integra LifeSciences Switzerland Sàrl, Le Locle, Switzerland) and Neurovent (RAUMEDIC AG, Helmbrechts, Germany) were the most frequently reported monitoring systems. Probe location and monitoring duration varied, and information on the device or probe placement was incomplete in several reports. A PbtO₂ threshold below 20 mmHg was the most commonly used definition of brain tissue hypoxia, although some studies used alternative thresholds, analysed PbtO₂ as a continuous variable, or derived oxygen-reactivity indices from the temporal relationship between PbtO₂ and systemic arterial pressure or cerebral perfusion pressure.

Seven reports were classified as having a high likelihood of overlap between patient populations, four as having possible overlap, and 10 as having no identified overlap. The principal potentially overlapping research programmes were based in Aachen, Columbia, Innsbruck, and Leipzig. Potentially overlapping reports were retained when they presented distinct exposures, interventions, analyses, or outcomes but were not interpreted as providing independent replication. Study characteristics and principal findings are summarised in Table 1.

**Table 1:** Characteristics and principal findings of the 21 included publication reports aSAH: aneurysmal subarachnoid haemorrhage; CBF: cerebral blood flow; CPP: cerebral perfusion pressure; CPPopt: optimal cerebral perfusion pressure; DCI: delayed cerebral ischaemia; FRx: flow-reactivity index; IAN: intra-arterial nimodipine; ICP: intracranial pressure; MAP: mean arterial pressure; NR: not reported; ORx: oxygen-reactivity index; PBrO₂/PbtO₂/PtiO₂: study-specific notation for invasive brain tissue oxygen tension; PRx: pressure-reactivity index

Study	Study design and setting	Total sample/PbtO₂ sample	Clinical context	Exposure/intervention	PbtO₂ monitoring	Principal finding	Cohort overlap
Raabe et al. (2005) [18]	Treatment-epoch analysis, Frankfurt/Leipzig, Germany	45/45	aSAH with cerebral vasospasm	Moderate/aggressive hypertension or hypervolaemia	Licox, white matter in parent-vessel territory	Moderate hypertension increased PbtO₂ in 90% of epochs, hypervolaemia in 12%	Possible (Frankfurt+Leipzig)
Jaeger et al. (2007) [27]	Continuous-monitoring cohort, Leipzig, Germany	67/67	aSAH with invasive monitoring	ORx and delayed cerebral infarction	Licox, frontal white matter	Higher ORx and lower day 5-6 PtiO₂ were associated with delayed cerebral infarction	Possible (Leipzig)
Al-Rawi et al. (2010) [37]	Prospective physiological intervention series, Cambridge, UK	44/44	Poor-grade aSAH	23.5% hypertonic saline	Neurotrend multiparameter probe	Hypertonic saline increased CPP, CBF, pH, and PbtO₂ and reduced ICP. A sustained response was associated with favourable outcome	None identified
Bhargava et al. (2011) [19]	Prospective pilot case series, UK centre	5/5	aSAH with delayed ischaemic deficit	Triple-H therapy	Invasive PtiO₂ with CBF and microdialysis	Feasibility was reported, but no extractable numerical PtiO₂ effect was provided	None identified
Hecht et al. (2011) [28]	Physiological case series, Berlin, Germany	5/5	aSAH with multimodal monitoring	CPP fluctuations and time-synchronised ORx/FRx	Licox plus thermal-diffusion CBF	PtiO₂ lagged CPP by 65±11 s. Time correction increased reactivity indices	None identified
Schmidt et al. (2011) [21]	Prospective cohort, Columbia, USA	30/30	Poor-grade aSAH	Spontaneous CPP variation and thresholds	Licox with ICP and microdialysis	CPP ≤70 mmHg was associated with hypoxia and metabolic crisis. Hypoxia was associated with death/severe disability	High likelihood (Columbia)
Schiefecker et al. (2013) [36]	Repeated-intervention analysis, Innsbruck, Austria	21/21	Poor-grade aSAH with fever	Parenteral diclofenac	Licox with ICP and microdialysis	PbtO₂ fell 13%; MAP and CPP fell about 10%. Some infusions precipitated hypoxia	Possible (Innsbruck)
Kurtz et al. (2014) [30]	Prospective repeated fluid-challenge study, Columbia, USA	10/10	aSAH undergoing fluid challenges	Cardiac-index responsiveness to fluid bolus	Invasive PbtO₂	PbtO₂ increased by 5.8 mmHg in cardiac-index responders vs. 1.3 mmHg in non-responders	High likelihood (Columbia)
Claassen et al. (2016) [29]	Retrospective time-series cohort, Columbia, USA	98/98	aSAH with multimodal physiology	Patient-specific causal relationships among systemic and cerebral variables	Licox with ICP and regional CBF	Systemic-PbtO₂ relationships were heterogeneous, patient-specific, and often bidirectional	High likelihood (Columbia)
Rass et al. (2019) [31]	Two-centre observational cohort, Innsbruck/Lausanne	100/100	Poor-grade aSAH	Protocolised PbtO₂ optimisation	Licox, frontal white matter in at-risk hemisphere	Target ≥20 mmHg was achieved during 75% of monitoring. Hypoxia remained common and was not independently associated with poor outcome	Possible (Innsbruck)
Fung et al. (2022) [34]	Prospective uncontrolled pilot, Bern, Switzerland	7/7	aSAH with DCI	Inhaled nitric oxide	Licox, frontal white matter	PtiO₂ increased at 19 of 29 time points. The uncontrolled design and increased catecholamine use limited inference	None identified
Narotam et al. (2022) [32]	Sequential case series, Omaha, USA	40/40	aSAH managed with brain-oxygen-directed protocol	Multicomponent PbtO₂-directed management	Invasive PbtO₂	Low-PbtO₂ burden fell from 67% to 15%. Mean PbtO₂ increased markedly after protocol implementation	None identified
Weiss et al. (2022) [23]	Before-after protocol comparison, Aachen, Germany	49/49	aSAH treated for DCI	Induced hypertension plus IAN vs. modified IAN protocol	Invasive PtiO₂	Modified protocol produced higher PtiO₂ and lower norepinephrine use, without significant outcome differences	High likelihood (Aachen)
Kastenholz et al. (2023) [25]	Retrospective analysis of prospective cohort, Aachen, Germany	76/76	aSAH with invasive monitoring	ORx and PRx, probe relation to hypoperfusion	Neurovent-PTO, frontal at-risk hemisphere	ORx was higher with the probe located within hypoperfused tissue. ORx and PRx were uncorrelated	High likelihood (Aachen)
Leppert et al. (2023) [33]	Retrospective before-after physiological study, Lübeck, Germany	30/30	aSAH with respiratory failure	Prone positioning	Invasive PBrO₂	Prone positioning improved oxygenation and PBrO₂ but increased ICP	None identified
Megjhani et al. (2023) [20]	Two-centre observational cohort, Columbia/Aachen	131/131	Poor-grade aSAH with multimodal monitoring	Measured CPP relative to individual CPPopt	Invasive PbtO₂, centre-specific placement	PbtO₂ was lower below CPPopt and plateaued at/above CPPopt (p<0.001)	High likelihood (Aachen+Columbia)
Olsen et al. (2023) [17]	Prospective physiological before-after study, Copenhagen, Denmark	36/17	Adults with aSAH within 5 days of ictus	Noradrenaline-induced MAP increase	Licox, intraparenchymal	MAP increased from 82 to 95 mmHg and PbtO₂ from 24 to 27 mmHg (p<0.001)	None identified
Moser et al. (2024) [35]	Retrospective repeated-dose analysis, Vienna, Austria	27/27	aSAH with multimodal monitoring	Oral nimodipine	Neurovent-PTO 2L with ICP/temperature	Small mean PbtO₂ fall after oral nimodipine. Critically low baseline values sometimes increased	None identified
Bele et al. (2025) [22]	Retrospective single-arm physiological series, Regensburg, Germany	6/6	aSAH with norepinephrine-refractory hypotension	Vasopressin bolus	Licox, intraparenchymal	MAP and CPP increased, and ICP decreased. The increase in PbtO₂ was transient and remained <20 mmHg	None identified
Carlson et al. (2025) [26]	Pooled observational cohort, New Mexico, USA	320/NR	aSAH with high-resolution multimodal monitoring	MAP variation and oxygen-based autoregulation	Licox in a subset, exact denominator NR	Worse ORx was associated with poorer outcome and increased before spreading-depolarisation clusters	None identified
Veldeman et al. (2026) [24]	Prospective cohort, Aachen, Germany	63/63	Unconscious aSAH patients treated for DCI	DCI treatment and multimodal response	Neurovent-PTO, ipsilateral/maximum blood load	PtiO₂ improved after DCI treatment. PtiO₂ change was not independently associated with 1-year outcome	High likelihood (Aachen)

Detailed study-level information on PbtO₂ definitions and monitoring characteristics, systemic cardiovascular, haemodynamic, and cardiorespiratory exposures, therapeutic interventions, temporal relationships, numerical findings, relevance to the review questions, methodological limitations, and potential cohort overlap is provided in Table 2.

**Table 2:** Detailed study-level extraction of brain tissue oxygenation in relation to systemic haemodynamic, cardiorespiratory, and therapeutic factors after aneurysmal subarachnoid haemorrhage Studies are presented chronologically by year of publication and alphabetically by first author within each year. Numbers in square brackets correspond to the numbered reference list. The publication report was the unit of inclusion. Reports from potentially overlapping cohorts should not be interpreted as independent patient samples. Relevance to the review question was classified descriptively according to the direction and nature of the relationship evaluated. These categories were used solely to organise the narrative synthesis and do not represent formal certainty-of-evidence or methodological-quality ratings. The physiological variables used to derive ORx varied among studies, and study-specific definitions are reported in the table. CBF: cerebral blood flow; CI: confidence interval; CPP: cerebral perfusion pressure; CPPopt: optimal cerebral perfusion pressure; CVP: central venous pressure; DCI: delayed cerebral ischaemia; DIND: delayed ischaemic neurological deficit; ECoG: electrocorticography; ETCO₂: end-tidal carbon dioxide; FRx: flow-reactivity index; HR: heart rate; IAN: intra-arterial nimodipine; ICP: intracranial pressure; IQR: interquartile range; LPR: lactate-to-pyruvate ratio; MAP: mean arterial pressure; NR: not reported; OR: odds ratio; ORx: oxygen-reactivity index; PaCO₂: arterial partial pressure of carbon dioxide; PaO₂: arterial partial pressure of oxygen; PBrO₂/PbtO₂/PtiO₂: study-specific notation for invasive brain tissue oxygen tension; ppm: parts per million; PRx: pressure-reactivity index; rCBF: regional cerebral blood flow; SD: spreading depolarisation; XeCT: xenon-enhanced computed tomography

Study	Design and PbtO₂ sample	Relevance to the review question, exposure, and temporal structure	PbtO₂ measurement and threshold	Key PbtO₂ finding	Cardiovascular/systemic interpretation	Main limitations and cohort overlap
Raabe et al. (2005) [18]	Treatment-epoch analysis, Frankfurt/Leipzig, Germany; total N/PbtO₂ n=45/45	Relevance: Indirect haemodynamic relevance. Exposure: Moderate induced hypertension, aggressive hypertension, or hypervolaemia. Temporal structure: 55 moderate-hypertension, 10 aggressive-hypertension, and 25 hypervolaemia periods	Licox in white matter in the parent-vessel territory. Impending ischaemia was defined as PbtO₂ <15 mmHg, with an intervention target >10 mmHg	Moderate hypertension increased PbtO₂ in 50 of 55 periods (90%), with a median increase of 6 mmHg (IQR: 3-10). Aggressive hypertension increased PbtO₂ in six of 10 periods (60%), with a median increase of 4 mmHg. Hypervolaemia increased PbtO₂ in three of 25 periods (12%), by 3-5 mmHg	Hypertension improved PbtO₂ more consistently than hypervolaemia. Treatment-related complications were reported but were not adjudicated as prespecified cardiac outcomes	Non-randomised treatment selection, non-independent epochs, historical therapy, and local probe placement. Cohort overlap: Possible (Frankfurt+Leipzig)
Jaeger et al. (2007) [27]	Continuous-monitoring cohort, Leipzig, Germany; total N/PbtO₂ n=67/67	Relevance: Contextual autoregulatory/prognostic relevance. Exposure: ORx, defined as the moving correlation between CPP and PtiO₂. Temporal structure: Delayed infarction (n=20) versus no infarction (n=47)	Licox in frontal white matter. Lower ORx values indicate more intact autoregulation, and no single PtiO₂ hypoxia threshold was used as the exposure	Mean ORx was 0.43±0.09 in patients with delayed infarction versus 0.23±0.14 in those without (p<0.0001). PtiO₂ was also lower on days 5-6 in patients with delayed infarction	Impaired oxygen reactivity and lower PtiO₂ were associated with delayed infarction, not with subsequent cardiac complications	Single-centre observational study with local probe placement. Cohort overlap: Possible (Leipzig component of Raabe et al. (2005) [18])
Al-Rawi et al. (2010) [37]	Prospective physiological intervention series, Cambridge, UK; total N/PbtO₂ n=44/44	Relevance: Indirect haemodynamic relevance. Exposure: 23.5% hypertonic saline, 2 mL/kg. Temporal structure: Within-patient baseline versus serial post-infusion time points	Neurotrend multiparameter probe. PbtO₂ response was assessed continuously, and no single hypoxia threshold was used as the primary endpoint	PbtO₂ increased significantly by 30 min and remained increased for up to 240 min. CPP, CBF, and pH increased, and ICP decreased. A sustained physiological response was associated with favourable outcome	Hypertonic saline was followed by improved cerebral perfusion and brain tissue oxygenation, but the uncontrolled design limited causal and cardiac inference	Uncontrolled intervention with repeat-dose and selection issues, an XeCT subgroup of n=16, and older monitoring technology. Cohort overlap: None identified
Bhargava et al. (2011) [19]	Prospective pilot case series, UK centre; total N/PbtO₂ n=5/5	Relevance: Indirect haemodynamic relevance, limited extractable data. Exposure: Triple-H therapy for delayed ischaemic neurological deficit. Temporal structure: Within-patient baseline, DIND onset, and response to therapy	Invasive PtiO₂ with CBF and microdialysis. Return of multimodal parameters to baseline defined treatment effectiveness	The full-text proceedings chapter reported feasibility and safety but did not provide an extractable numerical PtiO₂ effect	Triple-H therapy was relevant to systemic haemodynamics, but no numerical PtiO₂ effect was extractable	n=5. Preliminary full-text proceedings report with no numerical PtiO₂ effect. Device and recruitment details were not reported. Cohort overlap: None identified
Hecht et al. (2011) [28]	Physiological case series, Berlin, Germany; total N/PbtO₂ n=5/5	Relevance: Contextual autoregulatory relevance. Exposure: Spontaneous CPP fluctuations and modified time-synchronised ORx and FRx indices. Temporal structure: Conventional versus time-shift-corrected autoregulation indices	Licox plus thermal-diffusion CBF. No hypoxia threshold was used. PtiO₂ was used as a continuous autoregulation surrogate	The PtiO₂ response lagged CPP by 65±11 s, whereas the rCBF lag was 12±4 s. Time correction increased the reactivity indices	The time lag between CPP and PtiO₂ affected autoregulation indices. No cardiac endpoint was evaluated	n=5. Method-development study with no clinical outcome inference. Cohort overlap: None identified
Schmidt et al. (2011) [21]	Prospective cohort, Columbia, USA; total N/PbtO₂ n=30/30	Relevance: Indirect haemodynamic relevance. Exposure: CPP thresholds and spontaneous CPP variation. Temporal structure: Hours with CPP ≤70 versus >70 mmHg	Licox with ICP and microdialysis. Brain tissue hypoxia was defined as PbtO₂ <20 mmHg, and metabolic crisis was defined as LPR >40 plus glucose ≤0.7 mmol/L	CPP ≤70 mmHg was associated with brain tissue hypoxia (OR: 2.0; 95% CI: 1.2-3.3; p=0.007). Brain tissue hypoxia was also associated with death or severe disability	Lower CPP was associated with tissue hypoxia and metabolic crisis. The study did not evaluate cardiac dysfunction as an exposure or outcome	Small single-centre cohort, local probe, observational threshold analysis. Cohort overlap: High likelihood (Columbia)
Schiefecker et al. (2013) [36]	Repeated-intervention analysis, Innsbruck, Austria; total N/PbtO₂ n=21/21	Relevance: Indirect haemodynamic relevance. Exposure: Parenteral diclofenac 75 mg for fever control. Temporal structure: Pre-infusion baseline versus post-infusion time points	Licox with ICP and microdialysis. Brain tissue hypoxia was defined as PbtO₂ <20 mmHg	PbtO₂ decreased by 13%, from 28.1 to 24.5 mmHg (p<0.001). 43 of 123 infusions were followed by brain tissue hypoxia in eight of 21 patients	Diclofenac-associated decreases in MAP and CPP were accompanied by lower PbtO₂, supporting sensitivity to systemic haemodynamic changes	Non-randomised repeated infusions, small cohort, confounding by fever and concurrent care. Cohort overlap: Possible (Innsbruck component of Rass et al. (2019) [31])
Kurtz et al. (2014) [30]	Prospective repeated fluid-challenge study, Columbia, USA; total N/PbtO₂ n=10/10	Relevance: Direct cardiac-performance relevance. Exposure: Fluid bolus and cardiac-index response. Temporal structure: Cardiac-index responders (≥10% increase) versus non-responders	Invasive PbtO₂. PbtO₂ augmentation was assessed as a continuous and relative change	PbtO₂ increased by 5.8 mmHg (20.7%) in cardiac-index responders versus 1.3 mmHg (3.5%) in non-responders	This was the most direct cardiac link: PbtO₂ increased more when a fluid bolus increased cardiac index. The estimate was based on 10 patients and repeated, non-independent boluses	n=10 with 57 non-independent boluses and a very wide confidence interval. Cohort overlap: High likelihood (Columbia)
Claassen et al. (2016) [29]	Retrospective time-series cohort, Columbia, USA; total N/PbtO₂ n=98/98	Relevance: Indirect systemic cardiovascular relevance. Exposure: Patient-specific directional relationships among MAP, HR, CVP, ETCO₂, temperature, CPP, ICP, rCBF, and PbtO₂. Temporal structure: Early versus secondary post-haemorrhage phases, individualised causal models	Licox with ICP and regional CBF. The reference PbtO₂ target in the monitoring table was >15 mmHg	PbtO₂ showed patient-specific, model-derived directional relationships with cardiovascular and respiratory variables. CPP-PbtO₂ relationships were prominent	Systemic cardiovascular and respiratory variables showed heterogeneous, patient-specific relationships with PbtO₂. Temporal causality remained uncertain	Complex model-dependent inference, observational data, and variable availability. Cohort overlap: High likelihood (Columbia)
Rass et al. (2019) [31]	Two-centre observational cohort, Innsbruck/Lausanne; total N/PbtO₂ n=100/100	Relevance: Contextual multimodal physiological relevance. Exposure: Protocolised optimisation of CPP, PaCO₂, PaO₂, haemoglobin, temperature, sedation, and euvolaemia. Temporal structure: Periods with PbtO₂ <20 versus ≥20 mmHg, centre-specific protocols	Licox in frontal white matter in the at-risk hemisphere. Brain tissue hypoxia was defined as PbtO₂ <20 mmHg	The target PbtO₂ ≥20 mmHg was achieved during 75% of monitoring time. Hypoxia occurred in 81% of patients and during 25% of analysed time. Hypoxia was not independently associated with poor outcome	Brain tissue hypoxia frequently co-occurred with modifiable systemic abnormalities, but no single cardiac mechanism explained the episodes	Observational design, heterogeneous centre protocols, matched 2-h averages, local probe. Cohort overlap: Possible (Innsbruck component of Schiefecker et al. (2013) [36])
Fung et al. (2022) [34]	Prospective uncontrolled pilot study, Bern, Switzerland; total N/PbtO₂ n=7/7	Relevance: Contextual cardiorespiratory relevance. Exposure: Inhaled nitric oxide, titrated stepwise from 1 to 40 ppm. Temporal structure: Baseline versus post-titration and 4, 12, 24, and 48 h	Licox in frontal white matter. A positive PtiO₂ response was defined as an increase >5 mmHg	A positive PtiO₂ response occurred at 19 of 29 assessed time points. Median PtiO₂ was 27.7 mmHg at baseline, 27.3 immediately after titration, 33.0 at 4 h, 30.4 at 12 h, and 27.9 at 24 h	Inhaled nitric oxide was followed by variable PtiO₂ responses, while catecholamine support increased. Causal attribution was limited	No control group, very small, highly selected cohort, combined endpoint, concurrent rescue therapies. Cohort overlap: None identified
Narotam et al. (2022) [32]	Sequential case series, Omaha, USA; total N/PbtO₂ n=40/40	Relevance: Contextual multimodal physiological relevance. Exposure: Brain-oxygen-directed protocol comprising ICP control, eubaric hyperoxia, haemodynamic therapy, arterial vasodilation, and neuroprotection. Temporal structure: Serial phases after ictus and before and after protocol interventions	Invasive PbtO₂. Low brain oxygen was defined as PbtO₂ <20 mmHg	Low-PbtO₂ burden fell from 67% to 15%. Mean PbtO₂ increased from 11.31±9.34 to 27.85±6.76 mmHg (p<0.0001) and to 29.09±17.88 mmHg within 72 h	A multicomponent protocol was associated with increased PbtO₂, but the contributions of haemodynamic and non-haemodynamic components could not be separated	Non-randomised historical case series, complex multicomponent intervention, older single-centre data. Cohort overlap: None identified
Weiss et al. (2022) [23]	Before-after protocol comparison, Aachen, Germany; total N/PbtO₂ n=49/49	Relevance: Indirect haemodynamic relevance. Exposure: Classical induced-hypertension plus IAN protocol versus modified IAN-only protocol with a lower systolic arterial-pressure target. Temporal structure: 29 classical-protocol versus 20 modified-protocol patients	Invasive PtiO₂. Mean PtiO₂ during treatment was analysed, and no dichotomous threshold was used as the primary analysis	Mean PtiO₂ was higher with the modified protocol than with the classical protocol (39.6±15.4 versus 26.6±12.8 mmHg; p<0.01)	The lower-pressure protocol reduced noradrenaline exposure, while PtiO₂ remained higher. Comparative cardiovascular safety was not established	Non-randomised historical protocol comparison, refractory DCI, local IAN co-intervention. Cohort overlap: High likelihood (Aachen)
Kastenholz et al. (2023) [25]	Retrospective analysis of a prospective cohort, Aachen, Germany; total N/PbtO₂ n=76/76	Relevance: Contextual autoregulatory relevance. Exposure: Oxygen-reactivity index (ORx, CPP-PbtO₂) and pressure-reactivity index (PRx, MAP-ICP). Temporal structure: DCI versus no DCI, probe located inside versus outside a hypoperfused area	Neurovent-PTO in the frontal at-risk hemisphere. Brain tissue oxygen crisis was defined as PbtO₂ <20 mmHg	ORx was higher when the probe was located within a hypoperfused area (0.28±0.13 versus 0.18±0.15; p<0.05)	Probe location materially influenced ORx, limiting extrapolation from local oxygen reactivity to systemic cardiac physiology	Single-centre study, local probe, retrospective analysis. Cohort overlap: High likelihood (Aachen)
Leppert et al. (2023) [33]	Retrospective before-after physiological study, Lübeck, Germany; total N/PbtO₂ n=30/30	Relevance: Contextual cardiorespiratory relevance. Exposure: Prone positioning for respiratory insufficiency. Temporal structure: Supine baseline versus 1 h and 4 h during prone positioning	Invasive PBrO₂. No single PBrO₂ threshold was used as the primary result. ICP crises were assessed clinically	Prone positioning significantly increased arterial oxygenation and PBrO₂. A directly extractable numerical PBrO₂ effect estimate was not available	Prone positioning improved arterial oxygenation and PBrO₂ while increasing ICP. No cardiac endpoint was evaluated	Retrospective design, no non-prone control group, session-level repeated observations, device model and exact PBrO₂ effect not reported. Cohort overlap: None identified
Megjhani et al. (2023) [20]	Two-centre observational cohort, Columbia/Aachen; total N/PbtO₂ n=131/131	Relevance: Indirect haemodynamic relevance. Exposure: ΔCPP=measured CPP−individualised CPPopt. Temporal structure: CPP below, at, and above CPPopt, pre-DCI monitoring only	Invasive PbtO₂ with centre-specific probe placement. Brain tissue hypoxia was defined as PbtO₂ <20 mmHg	Brain tissue hypoxia occurred in 34.6% of hourly observations. PbtO₂ was lower when CPP was below CPPopt and reached a plateau at or above CPPopt (non-linear association; p<0.001)	The CPP-PbtO₂ relationship was non-linear and supported individualised CPP interpretation rather than indiscriminate pressure escalation	Observational two-centre data, local PbtO₂ measurement. Post-DCI data were excluded. The study links the Aachen and Columbia overlap clusters. Cohort overlap: High likelihood (Aachen+Columbia)
Olsen et al. (2023) [17]	Prospective physiological before-after study, Copenhagen, Denmark; total N/PbtO₂ n=36/17	Relevance: Indirect haemodynamic relevance. Exposure: Noradrenaline titration to increase MAP by up to 30 mmHg without exceeding 130 mmHg. Temporal structure: Within-patient baseline versus controlled arterial-pressure increase	Licox, intraparenchymal. No hypoxia threshold was required. Continuous PbtO₂ response was assessed	In the 17 monitored patients, PbtO₂ increased from 24 mmHg (95% CI: 19-31) to 27 mmHg (95% CI: 24-33; p<0.001)	Controlled MAP elevation produced a modest PbtO₂ increase. No cardiac outcome was evaluated	Small PbtO₂ subgroup, short intervention, surrogate physiological outcomes. Cohort overlap: None identified
Moser et al. (2024) [35]	Retrospective repeated-dose analysis, Vienna, Austria; total N/PbtO₂ n=27/27	Relevance: Indirect haemodynamic relevance. Exposure: Oral nimodipine 60 mg every 4 h or 30 mg every 2 h. Temporal structure: Pre-dose baseline versus serial post-dose measurements, dose-regimen comparison	Neurovent-PTO 2L with ICP and temperature. The clinical PbtO₂ goal was >20 mmHg. A subgroup with critical baseline values was analysed	PbtO₂ declined after approximately 50 min, with a maximum mean change of approximately −0.7 mmHg at 100 min. In patients with critical baseline values, PbtO₂ increased by up to 2.17 mmHg	Oral nimodipine slightly reduced MAP, CPP, and PbtO₂ overall, with divergent responses at critically low baseline PbtO₂	Retrospective repeated administrations, within-patient dependence. Small cohort, modest absolute effect. Cohort overlap: None identified
Bele et al. (2025) [22]	Retrospective single-arm physiological series, Regensburg, Germany; total N/PbtO₂ n=6/6	Relevance: Indirect haemodynamic relevance. Exposure: Vasopressin 3 IU bolus for noradrenaline-refractory hypotension. Temporal structure: Within-patient baseline versus 5, 10, 15, and 20 min	Licox, intraparenchymal. The clinical PbtO₂ target was >20 mmHg	PbtO₂ increased at 10 min (p<0.05) but did not reach ≥20 mmHg	Vasopressin increased MAP and CPP and transiently increased PbtO₂. The very short observation window precluded clinical cardiac inference	Retrospective design with no control group. The sample included six patients, with a very short observation window. Cohort overlap: None identified
Carlson et al. (2025) [26]	Pooled observational cohort, New Mexico, USA; total N/PbtO₂ n=320/not reported	Relevance: Contextual autoregulatory relevance. Exposure: Spontaneous MAP variation, ORx, and other autoregulation indices. Temporal structure: Clinical outcome strata and periods before and after isolated or clustered spreading depolarisations	Licox in a subset. The exact denominator was not reported. ORx was defined as the moving MAP-PbtO₂ correlation, and no single hypoxia threshold was used as the main exposure	Worse ORx was associated with worse clinical outcome (p=0.0005). ORx increased during the 60 min preceding spreading-depolarisation clusters (slope 0.0013; p=0.001)	MAP-linked oxygen-reactivity indices were associated with neurological outcomes, but the exact PbtO₂ subset and cardiac implications were unclear	Not every patient had every monitor. The exact PbtO₂ analytical n was not reported. Only 47 patients had ECoG, and the probe was often contralateral to tissue at risk. Cohort overlap: None identified
Veldeman et al. (2026) [24]	Prospective cohort, Aachen, Germany; total N/PbtO₂ n=63/63	Relevance: Indirect haemodynamic relevance. Exposure: Induced hypertension as first-line DCI treatment, endovascular rescue when refractory. Temporal structure: Pre-treatment versus post-treatment physiology, 1-year favourable versus unfavourable outcome	Neurovent-PTO, ipsilateral to the aneurysm or in the hemisphere with the greatest blood load. PtiO₂ was analysed continuously, and no hypoxia threshold was required for the primary outcome analysis	PtiO₂ improved after DCI treatment, but the change in PtiO₂ was not independently associated with 1-year outcome	PtiO₂ improved after DCI treatment, but treatment response did not independently predict outcome. Cardiac outcomes were not assessed	DCI-only, unconscious high-risk cohort, local PtiO₂ measurement. Cohort overlap: High likelihood (Aachen)

Evidence Addressing the Primary Review Question

None of the 21 included reports directly evaluated whether an antecedent abnormality in invasively measured brain tissue oxygen tension predicted a subsequent early cardiac complication or an episode of haemodynamic instability of cardiovascular origin [17-37]. No report assessed a temporally ordered association between PbtO₂, whether expressed as an absolute value, threshold-defined hypoxia, duration below a study-defined threshold, or cumulative hypoxic burden, and the subsequent occurrence of clinically significant arrhythmias, QTc prolongation, acute myocardial injury, new-onset left ventricular dysfunction, regional wall-motion abnormalities, stress cardiomyopathy, cardiogenic pulmonary oedema, or circulatory failure of cardiovascular origin.

The included reports instead examined contemporaneous associations between PbtO₂ and systemic arterial pressure, cerebral perfusion pressure, changes in cardiac index following fluid administration, respiratory variables, or vasoactive treatment, or immediate changes in PbtO₂ after physiological and therapeutic interventions. Accordingly, no direct effect estimate could be extracted, and no prognostic synthesis could be performed for the primary outcome.

Systemic Arterial Pressure, Cerebral Perfusion Pressure, and Cerebrovascular Autoregulation

A total of 12 reports provided directly extractable evidence on the relationships among systemic arterial pressure, cerebral perfusion pressure, vasoactive treatment, cerebrovascular autoregulation, and brain tissue oxygen tension [17,18,20-29]. A five-patient pilot case series evaluated multimodal monitoring during triple-H therapy but did not provide an extractable numerical estimate of the PtiO₂ response [19].

Controlled arterial pressure augmentation generally increased regional brain tissue oxygenation, although responses were variable. In the prospective study by Olsen et al., noradrenaline increased median mean arterial pressure from 82 mmHg (interquartile range (IQR): 76-85) to 95 mmHg (IQR: 88-98) and PbtO₂ from 24 mmHg (95% CI: 19-31) to 27 mmHg (95% CI: 24-33; p<0.001) among the 17 monitored patients [17]. Raabe et al. reported that moderate induced hypertension increased PbtO₂ in 50 of 55 treatment periods (90%), with a median increase of 6 mmHg (IQR: 3-10), whereas hypervolaemia increased PbtO₂ in only three of 25 periods [18].

The relationship between cerebral perfusion pressure and PbtO₂ was non-linear and patient-dependent. In a two-centre cohort of 131 patients, brain tissue hypoxia occurred in 34.6% of hourly observations. PbtO₂ was lower when measured cerebral perfusion pressure was below the individually estimated optimal cerebral perfusion pressure and reached a plateau at or above that value (p<0.001) [20]. In a separate cohort, cerebral perfusion pressure ≤70 mmHg was associated with brain tissue hypoxia (OR: 2.0; 95% CI: 1.2-3.3; p=0.007) and metabolic crisis (OR: 2.1; 95% CI: 1.2-3.7; p=0.007) [21]. In six patients with hypotension refractory to noradrenaline, vasopressin increased mean arterial pressure and cerebral perfusion pressure and reduced intracranial pressure, but the increase in PbtO₂ was transient, reached statistical significance only at 10 minutes, and did not restore PbtO₂ to ≥20 mmHg [22].

Protocol comparisons for delayed cerebral ischaemia also showed that higher systemic arterial pressure did not invariably result in superior PbtO₂ values or clinical outcomes. A modified protocol using intra-arterial nimodipine with a lower systolic arterial pressure target was associated with higher mean PtiO₂ than an earlier protocol combining induced hypertension with intra-arterial nimodipine (39.6±15.4 versus 26.6±12.8 mmHg; p<0.01) and lower noradrenaline requirements, without significant differences in delayed cerebral ischaemia-related infarction or in favourable outcome [23]. In a prospective cohort, PtiO₂ improved after delayed cerebral ischaemia treatment, but the magnitude of change was not independently associated with one-year functional outcome, whereas improvement in pressure reactivity was independently associated with favourable outcome [24].

PbtO₂-derived oxygen-reactivity indices provided additional evidence of regional and temporally dependent pressure-oxygen coupling. Oxygen reactivity was higher when the probe was located within a hypoperfused region than when it was located outside that region (0.28±0.13 versus 0.18±0.15; p<0.05), whereas oxygen reactivity and intracranial pressure-derived pressure reactivity were not correlated (r=−0.01; p=0.56) [25]. Worse oxygen reactivity was associated with poorer functional outcome and increased during the 60 minutes preceding clusters of spreading depolarisations [26]. In another cohort, mean oxygen reactivity was higher in patients who developed delayed cerebral infarction (0.43±0.09 versus 0.23±0.14; p<0.0001), and PtiO₂ during days 5-6 was lower in those who subsequently developed infarction (19.9±8.7 versus 26.3±7.6 mmHg; p=0.02) [27].

One five-patient series reported that PtiO₂ lagged behind changes in cerebral perfusion pressure by 65±11 seconds, compared with 12±4 seconds for regional cerebral blood flow. Adjusting for these delays increased the calculated reactivity indices [28]. A patient-specific time-series analysis reported heterogeneous and frequently bidirectional relationships between PbtO₂ and mean arterial pressure, cerebral perfusion pressure, central venous pressure, heart rate, end-tidal carbon dioxide, and regional cerebral blood flow [29].

Cardiac Output, Fluid Responsiveness, and Multimodal Physiological Optimisation

Direct evidence relating systemic blood flow or fluid responsiveness to brain tissue oxygenation was limited to one repeated fluid-challenge study, while two additional reports evaluated multicomponent PbtO₂-directed protocols [30-32]. In 10 patients receiving 57 fluid boluses, 27 boluses (47%) increased cardiac index by at least 10%. PbtO₂ increased by 5.8 mmHg (20.7%) after responsive boluses, compared with 1.3 mmHg (3.5%) after non-responsive boluses [30].

In a two-centre cohort of 100 patients managed according to a protocolised optimisation strategy targeting cerebral perfusion pressure, arterial oxygen and carbon dioxide tensions, haemoglobin, temperature, sedation, and euvolaemia, the PbtO₂ target of at least 20 mmHg was achieved during approximately 75% of the monitoring time. Nevertheless, 81% of patients experienced at least one hypoxic episode, with hypoxia accounting for 25% of the analysed monitoring time. During hypoxic observations, cerebral perfusion pressure <70 mmHg was present in 27%, arterial partial pressure of carbon dioxide (PaCO₂) <35 mmHg in 19%, arterial partial pressure of oxygen (PaO₂) <80 mmHg in 14%, haemoglobin <9 g/dL in 11%, metabolic crisis in 7%, and fever in 4%. PbtO₂ was not independently associated with poor outcome after multivariable adjustment (adjusted OR per 1 mmHg increase in PbtO₂: 0.98; 95% CI: 0.94-1.02; p=0.32) [31].

A sequential case series of 40 patients evaluated a broader brain-oxygen-directed protocol incorporating intracranial pressure control, eubaric hyperoxia, haemodynamic therapy, arterial vasodilation, and neuroprotective measures. The proportion of low-PbtO₂ measurements decreased from 67% to 15%, and the mean PbtO₂ increased from 11.31±9.34 to 27.85±6.76 mmHg following protocol implementation (p<0.0001) [32]. Because several therapeutic components were implemented simultaneously and no concurrent control group was available, their independent contributions could not be separated.

Respiratory and Gas-Exchange Interventions

Two reports primarily evaluated respiratory or gas-exchange interventions [33,34]. In 30 patients with aSAH and respiratory failure, prone positioning improved arterial oxygenation and increased PBrO₂ after one and four hours, although an exact numerical estimate of the PBrO₂ effect was not separately extractable. Intracranial pressure increased significantly, whereas cerebral perfusion pressure did not change significantly [33].

A seven-patient uncontrolled pilot study evaluated inhaled nitric oxide administered in stepwise doses of 1-40 ppm in patients with delayed cerebral ischaemia. A PtiO₂ response greater than 5 mmHg occurred at 19 of 29 assessed time points. The median PtiO₂ was 27.7 mmHg at baseline, 27.3 mmHg immediately after titration, 33.0 mmHg at four hours, 30.4 mmHg at 12 hours, and 27.9 mmHg at 24 hours. Systemic haemodynamic variables remained within 10% of baseline, although catecholamine requirements increased in all patients [34].

Additional respiratory evidence was derived from multimodal studies. Hypocapnia and arterial hypoxaemia were present during only a proportion of hypoxic observations in the protocolised optimisation cohort, while the fraction of inspired oxygen and arterial oxygen saturation contributed to PbtO₂ variation in the brain-oxygen-directed series [31,32]. Patient-specific modelling also demonstrated heterogeneous and occasionally bidirectional relationships between end-tidal carbon dioxide and PbtO₂ [29]. No included report evaluated controlled hypercapnia as an isolated intervention with an independently extractable PbtO₂ outcome. 

Pharmacological and Osmotic Interventions

Three reports evaluated oral nimodipine, intravenous diclofenac, or hypertonic saline in relation to brain tissue oxygenation [35-37]. Following oral nimodipine administration, mean arterial pressure and cerebral perfusion pressure decreased modestly, and the maximum mean reduction in PbtO₂ was approximately 0.7 mmHg at 100 minutes. Patients with critically low baseline PbtO₂ values showed increases of up to 2.17 mmHg. The 30 mg regimen produced a smaller reduction in mean arterial pressure and cerebral perfusion pressure than the 60 mg regimen but a similar PbtO₂ response [35].

Across 123 parenteral diclofenac infusions in 21 patients, mean PbtO₂ decreased by 13%, from 28.1 to 24.5 mmHg (p<0.001), while mean arterial pressure and cerebral perfusion pressure decreased by approximately 10%. Brain tissue hypoxia followed 43 infusions in eight patients, and cerebral perfusion pressure <70 mmHg was associated with hypoxia [36].

Administration of 23.5% hypertonic saline increased arterial pressure, cerebral perfusion pressure, cerebral blood flow velocity, brain pH, and PbtO₂ while reducing intracranial pressure. The increase in PbtO₂ was evident by 30 minutes and persisted for up to 240 minutes [37]. A response sustained beyond 210 minutes was associated with a favourable neurological outcome (p<0.023), although the uncontrolled design precluded attribution of an outcome benefit to the intervention. 

Neurological and Clinical Outcomes

Neurological outcomes were reported in several studies, but the association of PbtO₂ measures with these outcomes and their independent prognostic value were inconsistent [21,23,24,26,27,31,32,37]. Brain tissue hypoxia was associated with death or severe disability at three months in one cohort (OR: 5.1; 95% CI: 1.2-23.0; p=0.03), although the estimate was imprecise and comprehensive adjustment was not reported [21]. Worse oxygen reactivity was associated with poorer functional outcome and preceded clusters of spreading depolarisations in another pooled cohort [26], while higher oxygen reactivity and lower PtiO₂ during days 5-6 were associated with delayed cerebral infarction in the Leipzig cohort [27].

Other reports did not demonstrate an independent association between PbtO₂ and outcome. PbtO₂ was not independently associated with poor neurological outcome in the protocolised optimisation cohort [31], and improvement in PtiO₂ after delayed cerebral ischaemia treatment was not independently associated with one-year functional outcome [24]. Higher treatment-period PtiO₂ in the modified delayed cerebral ischaemia protocol did not translate into significant differences in delayed cerebral ischaemia-related infarction or favourable outcome [23]. In the multicomponent management series, coma at presentation, rather than a PbtO₂-derived measure, was the strongest reported predictor of mortality [32]. The inhaled nitric oxide pilot reported six-month outcomes but was not designed to estimate the effect of the intervention on clinical outcomes [34]. 

Critical Appraisal Findings

All 21 reports underwent design-specific critical appraisal using JBI instruments (Table 3) [17-37]. Cohort reports generally described exposures and physiological outcomes adequately, but confidence in their findings was reduced by residual confounding, incomplete adjustment for neurological severity and concurrent treatments, uncertainty regarding missing monitoring data, and the inclusion of highly selected populations. Many analyses examined contemporaneous relationships and therefore demonstrated physiological coupling rather than temporal prediction.

**Table 3:** Design-specific JBI critical appraisal findings for the 21 included publication reports The appraisal instrument was selected according to the study design and the analytical component that generated the evidence relevant to this review. Item-level appraisal was undertaken using the applicable design-specific JBI checklist. To facilitate comparison across heterogeneous study designs, item-level findings were organised descriptively into five overarching domains. The domain labels and summary judgements presented in this table were developed for this review and do not represent official JBI categories, validated risk-of-bias ratings, numerical scores, or thresholds for study inclusion. "Not applicable" indicates that the relevant appraisal item or domain was not applicable to the study's analytical design. Reports were not excluded on the basis of methodological quality. Potentially overlapping publication reports should not be interpreted as independent patient samples. DCI: delayed cerebral ischaemia; ECoG: electrocorticography; ICP: intracranial pressure; JBI: Joanna Briggs Institute; PbtO₂/PtiO₂: study-specific notation for invasive brain tissue oxygen tension; XeCT: xenon-enhanced computed tomography

Study	JBI tool	Selection/comparability	Exposure/measurement	Confounding/temporal trends	Outcome measurement/follow-up	Statistical analysis	Key methodological limitations
Raabe et al. (2005) [18]	JBI quasi-experimental tool (2024)	Important concerns	Adequately addressed	Important concerns	Adequately addressed	Some concerns	Non-randomised treatment selection, differing co-interventions, non-independent treatment epochs, historical therapeutic context, and possible Leipzig overlap
Jaeger et al. (2007) [27]	JBI cohort tool (2025)	Adequately addressed	Adequately addressed	Some concerns	Some concerns	Adequately addressed	Single-centre study with local probe sampling, incomplete reporting of confounding control and data completeness, and possible Leipzig overlap
Al-Rawi et al. (2010) [37]	JBI quasi-experimental tool (2024)	Some concerns	Adequately addressed	Important concerns	Adequately addressed	Adequately addressed	Uncontrolled intervention, possible confounding related to repeat dosing and participant selection, and a smaller XeCT subgroup
Bhargava et al. (2011) [19]	JBI case series tool (2020)	Some concerns	Adequately addressed	Some concerns	Some concerns	Some concerns	Very small preliminary report with limited reporting of recruitment, participant characteristics, outcomes, and statistical methods
Hecht et al. (2011) [28]	JBI case series tool (2020)	Some concerns	Adequately addressed	Some concerns	Adequately addressed	Some concerns	Five-patient method-development report with uncertain case capture and limited reporting of participant characteristics and statistical methods
Schmidt et al. (2011) [21]	JBI cohort tool (2025)	Adequately addressed	Adequately addressed	Adequately addressed	Adequately addressed	Adequately addressed	Small single-centre observational threshold analysis, local probe sampling, and a high likelihood of Columbia cohort overlap
Schiefecker et al. (2013) [36]	JBI quasi-experimental tool (2024)	Some concerns	Adequately addressed	Important concerns	Adequately addressed	Adequately addressed	Non-randomised repeated infusions, no concurrent control group, confounding by fever and rescue care, and possible Innsbruck overlap
Kurtz et al. (2014) [30]	JBI quasi-experimental tool (2024)	Adequately addressed	Adequately addressed	Important concerns	Adequately addressed	Adequately addressed	Only 10 patients contributed 57 non-independent boluses. There was no untreated control group, and the estimates were imprecise
Claassen et al. (2016) [29]	JBI cohort tool (2025)	Not applicable to the analytical design	Adequately addressed	Adequately addressed	Some concerns	Adequately addressed	Complex model-dependent observational inference, variable signal availability, and a high likelihood of Columbia cohort overlap
Rass et al. (2019) [31]	JBI cohort tool (2025)	Some concerns	Adequately addressed	Important concerns	Adequately addressed	Adequately addressed	Two-centre protocol heterogeneity, mainly contemporaneous exposure-outcome associations, local probe sampling, and possible Innsbruck overlap
Fung et al. (2022) [34]	JBI quasi-experimental tool (2024)	Important concerns	Adequately addressed	Important concerns	Adequately addressed	Some concerns	Very small uncontrolled pilot, highly selected DCI population, composite physiological response definition, and concurrent rescue treatments
Narotam et al. (2022) [32]	JBI case series tool (2020)	Some concerns	Adequately addressed	Adequately addressed	Adequately addressed	Some concerns	Historical, non-randomised case series with a multicomponent intervention and uncertain consecutive or complete case capture
Weiss et al. (2022) [23]	JBI quasi-experimental tool (2024)	Important concerns	Adequately addressed	Some concerns	Adequately addressed	Adequately addressed	Non-randomised historical comparison with a local intra-arterial co-intervention, residual calendar-time confounding, and a high likelihood of Aachen overlap
Kastenholz et al. (2023) [25]	JBI cohort tool (2025)	Adequately addressed	Adequately addressed	Adequately addressed	Adequately addressed	Adequately addressed	Retrospective single-centre analysis with local probe sampling and a high likelihood of overlap with other Aachen reports
Leppert et al. (2023) [33]	JBI quasi-experimental tool (2024)	Some concerns	Adequately addressed	Important concerns	Adequately addressed	Some concerns	Retrospective uncontrolled session-level analysis of 97 sessions in 30 patients, with potential confounding by ventilator and ICP interventions
Megjhani et al. (2023) [20]	JBI cohort tool (2025)	Some concerns	Adequately addressed	Adequately addressed	Some concerns	Adequately addressed	Two-centre observational data, monitor-specific missingness, local probe sampling, and overlap with the Aachen and Columbia cohorts
Olsen et al. (2023) [17]	JBI quasi-experimental tool (2024)	Adequately addressed	Adequately addressed	Important concerns	Adequately addressed	Adequately addressed	Small PbtO₂ subgroup (17/36), no concurrent control group, brief exposure, and surrogate physiological outcomes
Moser et al. (2024) [35]	JBI quasi-experimental tool (2024)	Some concerns	Adequately addressed	Important concerns	Adequately addressed	Adequately addressed	Retrospective repeated-dose analysis, no untreated control group, residual confounding by concurrent care, and a modest absolute PbtO₂ change
Bele et al. (2025) [22]	JBI quasi-experimental tool (2024)	Adequately addressed	Adequately addressed	Important concerns	Adequately addressed	Some concerns	Retrospective single-arm study, n=6, no concurrent control group, and a 20-minute physiological observation window.
Carlson et al. (2025) [26]	JBI cohort tool (2025)	Adequately addressed	Adequately addressed	Some concerns	Some concerns	Adequately addressed	Monitor availability varied, the exact PbtO₂ denominator was unclear, and probe and ECoG placement could introduce measurement-selection bias
Veldeman et al. (2026) [24]	JBI cohort tool (2025)	Adequately addressed	Adequately addressed	Adequately addressed	Important concerns	Adequately addressed	High-risk DCI-only cohort, local PtiO₂ sampling, incomplete 1-year outcome data, and a high likelihood of Aachen cohort overlap

The quasi-experimental evidence consisted predominantly of uncontrolled before-after studies, single-arm physiological investigations, and treatment-epoch analyses. Most lacked a concurrent control group and were vulnerable to confounding by disease progression, simultaneous interventions, and changes in clinical management. Several reports analysed repeated boluses, infusions, or monitoring epochs within the same patients as separate observations without fully accounting for within-patient dependence, thereby limiting statistical independence. The case-series reports were limited by small sample sizes, incomplete reporting of participant selection, the absence of comparators, and multicomponent interventions.

Across study designs, PbtO₂ provided an objective but regional measure of cerebral tissue oxygen tension. Variation in probe location, monitoring device, stabilisation procedures, hypoxia thresholds, data processing methods, and the duration of valid monitoring introduced measurement heterogeneity. Potential cohort overlap further limited the interpretation of concordant findings as independent replication. Collectively, these methodological limitations restricted causal inference and precluded firm conclusions regarding comparative treatment effectiveness or the independent prognostic value of PbtO₂.

Discussion

Principal Findings

This systematic review included 21 publication reports examining relationships between invasively measured brain tissue oxygen tension and systemic haemodynamic, cardiorespiratory, or therapeutic factors in adults with aSAH [17-37]. None assessed whether an antecedent PbtO₂ abnormality or cumulative hypoxic burden predicted a subsequent early cardiac complication. The prognostic relationships with arrhythmias, QTc prolongation, myocardial injury, ventricular dysfunction, stress cardiomyopathy, cardiogenic pulmonary oedema, and cardiac-related haemodynamic instability therefore remain unresolved.

Most reports addressed reverse or contemporaneous relationships. Lower mean arterial pressure or cerebral perfusion pressure was often associated with lower PbtO₂, and arterial pressure augmentation increased PbtO₂ in several studies. However, responses varied with baseline oxygenation, autoregulatory status, probe location, local pathology, and the relationship between measured cerebral perfusion pressure and an individual physiological optimum [17-29]. These findings do not support a universal mean arterial pressure or cerebral perfusion pressure target.

Evidence on systemic blood flow was limited to a small fluid-challenge study and multicomponent protocols [30-32]. A greater PbtO₂ increase occurred when fluid administration increased cardiac index, but this does not establish a cardiac index target. Respiratory, pharmacological, and osmotic interventions also produced variable PbtO₂ changes, often alongside simultaneous changes in systemic, cerebral, or intracranial physiology [33-37].

Associations between PbtO₂ or oxygen-reactivity measures and metabolic crisis, spreading depolarisation, delayed cerebral infarction, mortality, or neurological outcome were inconsistent and were not uniformly independent of disease severity or concurrent physiological disturbances [21,23,24,26,27,31,32,37]. PbtO₂ is therefore best regarded as a regional physiological signal rather than an established standalone prognostic biomarker.

Interpretation and Clinical Implications

The absence of direct evidence for the primary review question reflects the absence of temporally ordered prognostic studies, not evidence that brain tissue oxygenation and cardiac complications are biologically unrelated. Cardiac dysfunction may reduce cerebral perfusion and oxygen delivery and thereby lower PbtO₂, whereas severe cerebral injury may provoke catecholamine-mediated myocardial injury and electrical or ventricular dysfunction [4-6]. Contemporaneous associations cannot distinguish prediction from reverse causation or from the common influence of haemorrhage severity.

Interpretation must also account for autoregulation and regional sampling. A PbtO₂ probe measures a small volume of surrounding tissue. A low value may reflect focal hypoperfusion, vasospasm, local injury, increased metabolic demand, or impaired systemic oxygen delivery, while a normal value cannot exclude hypoxia elsewhere. Probe location, imaging findings, signal validity, recent interventions, and the proportion of technically valid data should therefore be reviewed.

Clinically, low PbtO₂ should prompt a structured search for reversible contributors, including inadequate mean arterial pressure or cerebral perfusion pressure, elevated intracranial pressure, hypoxaemia, hypocapnia, anaemia, fever, impaired cardiac output or ventricular function, vasospasm, and delayed cerebral ischaemia. Treatment should address the most plausible mechanism, and the response should be reassessed using PbtO₂ together with mean arterial pressure, cerebral perfusion pressure, intracranial pressure, arterial blood gases, haemoglobin, temperature, cardiac performance, and the broader clinical context. Routine pressure augmentation, fluid loading, or respiratory manipulation based solely on a single PbtO₂ threshold is not supported, and any cerebral benefit must be balanced against cardiovascular, pulmonary, and intracranial harm.

Current evidence does not support PbtO₂ as a standalone predictor of early cardiac complications or as a replacement for standard cardiac surveillance and clinically indicated cardiovascular assessment. Its present clinical value lies in detecting regional cerebral oxygen disturbances and evaluating physiological responses to selected interventions, particularly when neurological examination is unreliable.

Comparison With Previous Reviews and Current Guidance

The findings are consistent with contemporary aSAH guidance, which supports individualised haemodynamic management and selective advanced neuromonitoring while acknowledging the low certainty of much of the evidence [1,38]. The 2023 American Heart Association/American Stroke Association guideline discusses invasive brain tissue oxygen and metabolic monitoring in selected patients with severe aSAH but does not establish routine PbtO₂-guided treatment or a universal threshold. It supports euvolaemia and blood pressure elevation for symptomatic delayed cerebral ischaemia while advising against prophylactic haemodynamic augmentation and hypervolaemia [1].

Previous evidence syntheses reported that thresholds of 15-20 mmHg are commonly used to define brain tissue hypoxia and that lower PbtO₂ may be associated with delayed cerebral ischaemia [10,39,40]. Reviews of multimodal or systemic haemodynamic monitoring likewise found heterogeneous, predominantly low-certainty evidence and no consistent improvement in patient-centred outcomes [11,39]. These findings support physiological plausibility but not a universally applicable threshold or proof that correcting PbtO₂ improves outcome.

The present review extends this literature by separating immediate or contemporaneous effects of systemic haemodynamic and cardiorespiratory factors on PbtO₂ from the distinct prognostic question of whether antecedent PbtO₂ abnormalities predict subsequent cardiac complications. The absence of evidence for the latter identifies a specific unresolved research gap.

Strengths and Limitations

This review addressed a clinically specific and previously underexplored question by separating two related but methodologically distinct directions of the cardiocerebral relationship after aSAH: whether abnormalities in brain tissue oxygenation predict subsequent early cardiac complications and how systemic haemodynamic or cardiorespiratory factors influence PbtO₂. Eligibility was restricted to invasive intraparenchymal monitoring, and predefined operational definitions were used for brain tissue hypoxia, cardiac complications, temporal prediction, and haemodynamic instability.

Multiple bibliographic databases and a trial registry were searched without date or language restrictions, and the searches were updated on 10 October 2026. Backward and forward citation searching were performed, design-specific JBI instruments were used, and potential cohort overlap was considered. The synthesis preserved the exposure definitions, thresholds, analytical units, and measurement intervals of the original reports and did not combine clinically or methodologically incompatible data solely to permit pooling.

The principal limitation is that no included report directly addressed the primary prognostic question. The available evidence consisted mainly of observational cohorts, uncontrolled physiological studies, before-after analyses, and small case series. No randomised controlled trial met the eligibility criteria. Repeated interventions or monitoring epochs were sometimes analysed within the same participants, and residual confounding, temporal ambiguity, treatment-selection bias, and simultaneous co-interventions limited causal interpretation.

Clinical and methodological heterogeneity was substantial, including differences in disease severity, monitoring indications, probe location, hypoxia thresholds, monitoring duration, data-cleaning procedures, interventions, analytical units, and outcome definitions. The regional nature of PbtO₂ and possible overlap between institutional cohorts further limited interpretation. These factors precluded meta-analysis, formal subgroup analysis, assessment of small-study effects, and formal certainty-of-evidence grading.

No protocol was prospectively registered, and study selection, data extraction, and critical appraisal were performed by a single reviewer, increasing the risk of selection, extraction, and appraisal error. Seventeen potentially relevant reports could not be retrieved through the available legal access routes and could not undergo full eligibility assessment. Availability bias cannot be excluded. Some accessible reports had incomplete numerical data, unclear denominators, or limited reporting of missing physiological data. Forward citation searching used a single citation index, OpenAlex, so incompletely indexed citations may have been missed. Although all database and registry searches were updated on 10 October 2026, database contents and indexing continue to change. The conclusions should therefore be interpreted as describing physiological patterns, methodological limitations, and evidence gaps rather than establishing causal relationships, universal thresholds, or validated treatment targets.

Implications for Future Research

Prospective multicentre studies should determine whether PbtO₂ abnormalities precede and independently predict early cardiac complications after aSAH. The cerebral exposure window should be prespecified and should end before the adjudicated cardiac outcome, with synchronised cerebral, systemic, and cardiac monitoring to establish temporal direction and reduce reverse-causation bias.

PbtO₂ exposure should be characterised using standardised time-dependent measures, including episode frequency and duration, the proportion of valid monitoring time below a predefined threshold, and cumulative or time-weighted hypoxic burden. Probe location, signal quality, artefact handling, missing-data procedures, and relevant cardiac outcomes should be reported consistently. Analyses should account for repeated measurements and time-varying treatment and should adjust for major neurological, cardiovascular, respiratory, and treatment-related confounders.

Interventional trials should be considered only after reproducible observational evidence identifies temporally valid and clinically actionable targets. Such trials should assess neurological, cardiac, respiratory, functional, and safety outcomes rather than short-term changes in PbtO₂ alone.

## Conclusions

No included report directly assessed whether antecedent PbtO₂ abnormalities or cumulative hypoxic burden predict subsequent early cardiac complications after aSAH. Current evidence instead shows that PbtO₂ varies with systemic arterial pressure, cerebral perfusion pressure, autoregulatory status, effective systemic blood flow, respiratory physiology, intracranial conditions, and probe-related factors. PbtO₂ should therefore be interpreted as a regional component of multimodal neuromonitoring rather than as a standalone cardiac prognostic biomarker or universal haemodynamic treatment target. Prospective studies with synchronised cerebral and cardiovascular monitoring are required.

## Appendices

Appendix A

The following table provides the complete database-specific search strategies.

**Table 4:** Complete database-specific search strategies Searches had no publication-date or language restrictions. Counts are raw before deduplication. Database and registry updates on 10 October 2026 yielded no additional records. The Scopus count includes one internal duplicate removed during deduplication. Forward citation searching identified 808 records: 93 matched the existing dataset, and 715 additional records were screened. No new eligible report was identified. Counts may change as databases are updated. CENTRAL: Cochrane Central Register of Controlled Trials; PbtO2: brain tissue oxygen tension; SAH: subarachnoid haemorrhage; aSAH: aneurysmal subarachnoid haemorrhage

Information source and search details	Complete search strategy
Source: MEDLINE via PubMed	("Subarachnoid Hemorrhage"[Mesh] OR "subarachnoid hemorrhage"[tiab] OR "subarachnoid haemorrhage"[tiab] OR "aneurysmal subarachnoid hemorrhage"[tiab] OR "aneurysmal subarachnoid haemorrhage"[tiab] OR aSAH[tiab] OR SAH[tiab]) AND ("brain tissue oxygen"[tiab] OR "brain tissue oxygenation"[tiab] OR "brain tissue oxygen tension"[tiab] OR "brain tissue oxygen pressure"[tiab] OR "brain tissue oxygen partial pressure"[tiab] OR "partial pressure of brain tissue oxygen"[tiab] OR "brain oxygen tension"[tiab] OR "brain tissue oximetry"[tiab] OR "intraparenchymal oxygen"[tiab] OR PbtO2[tiab] OR "PbtO(2)"[tiab] OR PbO2[tiab] OR PbrO2[tiab] OR PtiO2[tiab] OR "PtiO(2)"[tiab] OR Licox[tiab] OR "Neurovent-PTO"[tiab] OR "Neurovent PTO"[tiab])
Search date: 10 October 2026	
Records: 221	
Source: Web of Science	TS=("subarachnoid hemorrhage" OR "subarachnoid haemorrhage" OR "aneurysmal subarachnoid hemorrhage" OR "aneurysmal subarachnoid haemorrhage" OR aSAH) AND TS=("brain tissue oxygen tension" OR "brain tissue oxygen pressure" OR "brain tissue oxygenation" OR "brain tissue oxygen" OR PbtO2 OR PtiO2 OR PbO2 OR PbrO2 OR "pti-pO2")
Search date: 10 October 2026	
Records: 271	
Source: Scopus	TITLE-ABS-KEY("subarachnoid hemorrhage" OR "subarachnoid haemorrhage" OR "aneurysmal subarachnoid hemorrhage" OR "aneurysmal subarachnoid haemorrhage" OR aSAH) AND TITLE-ABS-KEY("brain tissue oxygen tension" OR "brain tissue oxygen pressure" OR "brain tissue oxygenation" OR "brain tissue oxygen" OR PbtO2 OR PtiO2 OR PbO2 OR PbrO2 OR "pti-pO2")
Search date: 10 October 2026	
Records: 263	
Source: CENTRAL	#1 MeSH descriptor: [Subarachnoid Hemorrhage] explode all trees #2 ("subarachnoid hemorrhage" OR "subarachnoid haemorrhage" OR "aneurysmal subarachnoid hemorrhage" OR "aneurysmal subarachnoid haemorrhage" OR aSAH):ti,ab,kw #3 ("brain tissue oxygen tension" OR "brain tissue oxygen pressure" OR "brain tissue oxygenation" OR "brain tissue oxygen" OR PbtO2 OR PtiO2 OR PbO2 OR PbrO2 OR "pti-pO2"):ti,ab,kw #4 (#1 OR #2) AND #3
Search date: 10 October 2026	
Records: 10	
Source: Europe PMC	(TITLE_ABS:"subarachnoid hemorrhage" OR TITLE_ABS:"subarachnoid haemorrhage" OR TITLE_ABS:"aneurysmal subarachnoid hemorrhage" OR TITLE_ABS:"aneurysmal subarachnoid haemorrhage" OR TITLE_ABS:aSAH) AND (TITLE_ABS:"brain tissue oxygen tension" OR TITLE_ABS:"brain tissue oxygen pressure" OR TITLE_ABS:"brain tissue oxygenation" OR TITLE_ABS:"brain tissue oxygen" OR TITLE_ABS:PbtO2 OR TITLE_ABS:PtiO2 OR TITLE_ABS:PbO2 OR TITLE_ABS:PbrO2 OR TITLE_ABS:"pti-pO2")
Search date: 10 October 2026	
Records: 173	
Source: ClinicalTrials.gov	Condition: ("subarachnoid hemorrhage" OR "subarachnoid haemorrhage" OR "aneurysmal subarachnoid hemorrhage" OR "aneurysmal subarachnoid haemorrhage" OR aSAH) Other terms: ("brain tissue oxygen" OR "brain tissue oxygenation" OR PbtO2 OR PtiO2 OR PbrO2 OR PbO2 OR "pti-pO2")
Search date: 10 October 2026	
Records: 22	
Source: Backward citation searching	The reference lists of potentially eligible reports and relevant evidence syntheses were examined manually. One additional unique report was identified.
Search date: 24 July 2026	
Records: 1	
Source: Forward citation searching	All 21 included publication reports were used as seed reports in OpenAlex. Records indexed as citing at least one seed report were retrieved using the cites relationship, with publication dates up to 10 October 2026. The union contained 808 records. After deduplication against the existing review dataset, 715 additional records underwent title and abstract screening. No additional eligible report was identified.
Search date: 10 October 2026	
Records: 808	

Appendix B

The following table provides the details of the reports sought for retrieval but not obtained in full text during the review process.

**Table 5:** Reports sought for retrieval but not obtained in full text Bibliographic identifiers were checked against PubMed and/or the publisher record. "Report not retrieved" means that the full text was not obtained during the review's documented retrieval process, despite attempts through legally accessible publisher, bibliographic, institutional, and author-sharing sources. It does not mean that no bibliographic record or abstract exists. DOI letter case is not significant. PRISMA: Preferred Reporting Items for Systematic Reviews and Meta-Analyses

No.	First author	Year	Article title	DOI	PMID	PRISMA status
1	Ianosi B	2020	An Observational Study on the Use of Intravenous Non-Opioid Analgesics and Antipyretics in Poor-Grade Subarachnoid Hemorrhage: Effects on Hemodynamics and Systemic and Brain Temperature.	10.1089/ther.2018.0046	30835164	Report not retrieved
2	Gaasch M	2018	Cerebral Autoregulation in the Prediction of Delayed Cerebral Ischemia and Clinical Outcome in Poor-Grade Aneurysmal Subarachnoid Hemorrhage Patients.	10.1097/CCM.0000000000003016	29394184	Report not retrieved
3	Hockel K	2017	Effect of Intra-Arterial and Intravenous Nimodipine Therapy of Cerebral Vasospasm After Subarachnoid Hemorrhage on Cerebrovascular Reactivity and Oxygenation.	10.1016/j.wneu.2017.02.014	28232152	Report not retrieved
4	Titova IV	2013	[Influence of cerebral perfusion pressure and cardiac output on brain oxygenation and metabolism].	No DOI indexed	24341044	Report not retrieved
5	Bohman LE	2013	Response of brain oxygen to therapy correlates with long-term outcome after subarachnoid hemorrhage.	10.1007/s12028-013-9890-6	23949477	Report not retrieved
6	Choi HA	2012	Acute effects of nimodipine on cerebral vasculature and brain metabolism in high grade subarachnoid hemorrhage patients.	10.1007/s12028-012-9670-8	22262041	Report not retrieved
7	Dengl M	2012	Comparing brain tissue oxygen measurements and derived autoregulation parameters from different probes (Licox vs. Raumedic).	10.1007/978-3-7091-0956-4_31	22327685	Report not retrieved
8	Jaeger M	2012	Clinical significance of impaired cerebrovascular autoregulation after severe aneurysmal subarachnoid hemorrhage.	10.1161/STROKEAHA.112.659888	22618384	Report not retrieved
9	Stuart RM	2011	High-dose intra-arterial verapamil for the treatment of cerebral vasospasm after subarachnoid hemorrhage: prolonged effects on hemodynamic parameters and brain metabolism.	10.1227/NEU.0b013e318201be47	21135735	Report not retrieved
10	Barth M	2010	Correlation of Clinical Outcome with Pressure-, Oxygen-, and Flow-Related Indices of Cerebrovascular Reactivity in Patients Following Aneurysmal SAH.	10.1007/s12028-009-9287-8	19816810	Report not retrieved
11	Muench E	2007	Effects of hypervolemia and hypertension on regional cerebral blood flow, intracranial pressure, and brain tissue oxygenation after subarachnoid hemorrhage.	10.1097/01.CCM.0000275392.08410.DD	17581487	Report not retrieved
12	Muench E	2005	Effects of positive end-expiratory pressure on regional cerebral blood flow, intracranial pressure, and brain tissue oxygenation.	10.1097/01.CCM.0000181732.37319.DF	16215394	Report not retrieved
13	Al-Rawi PG	2005	Cerebral blood flow augmentation in patients with severe subarachnoid haemorrhage.	10.1007/3-211-32318-X_27	16463835	Report not retrieved
14	Stiefel MF	2004	The effect of nimodipine on cerebral oxygenation in patients with poor-grade subarachnoid hemorrhage.	10.3171/jns.2004.101.4.0594	15481712	Report not retrieved
15	Reinprecht A	2003	Prone position in subarachnoid hemorrhage patients with acute respiratory distress syndrome: effects on cerebral tissue oxygenation and intracranial pressure.	10.1097/01.CCM.0000063453.93855.0A	12794427	Report not retrieved
16	Väth A	2002	Therapeutic aspects of brain tissue pO2 monitoring after subarachnoid hemorrhage.	10.1007/978-3-7091-6738-0_78	12168333	Report not retrieved
17	Kett-White R	2002	Cerebral oxygen and microdialysis monitoring during aneurysm surgery: effects of blood pressure, cerebrospinal fluid drainage, and temporary clipping on infarction.	10.3171/jns.2002.96.6.1013	12066900	Report not retrieved
